# A Generalized Polynomial Chaos-Based Approach to Analyze the Impacts of Process Deviations on MEMS Beams

**DOI:** 10.3390/s17112561

**Published:** 2017-11-08

**Authors:** Lili Gao, Zai-Fa Zhou, Qing-An Huang

**Affiliations:** Key Laboratory of MEMS of the Ministry of Education, Southeast University, Nanjing 210096, China; LilyGaoChina@gmail.com

**Keywords:** MEMS beams, GaAs MMIC-based process, stochastic process deviations, GPC, MC

## Abstract

A microstructure beam is one of the fundamental elements in MEMS devices like cantilever sensors, RF/optical switches, varactors, resonators, etc. It is still difficult to precisely predict the performance of MEMS beams with the current available simulators due to the inevitable process deviations. Feasible numerical methods are required and can be used to improve the yield and profits of the MEMS devices. In this work, process deviations are considered to be stochastic variables, and a newly-developed numerical method, i.e., generalized polynomial chaos (GPC), is applied for the simulation of the MEMS beam. The doubly-clamped polybeam has been utilized to verify the accuracy of GPC, compared with our Monte Carlo (MC) approaches. Performance predictions have been made on the residual stress by achieving its distributions in GaAs Monolithic Microwave Integrated Circuit (MMIC)-based MEMS beams. The results show that errors are within 1% for the results of GPC approximations compared with the MC simulations. Appropriate choices of the 4-order GPC expansions with orthogonal terms have also succeeded in reducing the MC simulation labor. The mean value of the residual stress, concluded from experimental tests, shares an error about 1.1% with that of the 4-order GPC method. It takes a probability around 54.3% for the 4-order GPC approximation to attain the mean test value of the residual stress. The corresponding yield occupies over 90 percent around the mean within the twofold standard deviations.

## 1. Introduction

Numerical simulation methods have widely been used in the design of Micro-Electromechanical Systems (MEMS) to model the interactions among multi-physical fields for rapid computational prototyping [1,2,3,4]. However, owing to factors like manufacturing process errors, residual stresses, irregular surface topography, and chemical contamination, these simulation methods assume the geometrical and physical properties of the device to be determinate [5]. Ubiquitous uncertainty exists for incomplete underlying physics theories and inevitable measurement errors. To investigate the impact of data drifts in processing, the quantification of uncertainty needs to be developed. Therefore, for reliable predictions, it is imperative to incorporate uncertainty when the simulations begin, not as an after-thought [6,7]. The stochastic deviations in various design parameters should be considered during the development of the computational models.

MEMS uncertainties have been considered to be subjective safety factors, which may lead to over conservative designs [8]. The influence of processing uncertainties on the operation and reliability of MEMS devices has been widely investigated on both the experimental and the theoretical points of view. Although improvements have been achieved in single processing steps like release [9] and surface roughness [10], or in topology and material optimizations [11,12,13], MEMS device optimization through various experimental trials is time-profit costing. On the other hand, computer-aided designs or simulations can quickly provide proper guidance for device design when facing process deviations. Typically, Monte Carlo (MC) simulations have been employed to deal with the input parameter uncertainty during MEMS device design [14,15,16,17]. These studies have presented natural but expensive MC-based frameworks in a deterministic way. Even if it is straightforward, the MC method becomes prohibitively expensive to achieve high accuracy as it offers slow convergence rates when facing complex multi-physics MEMS problems. Although improvements have been made by researchers [18,19], the heavy sampling remains an obstacle. In other cases where the problem is entirely continuous, Taylor expansions seem to be useful [20]. Unfortunately, it is just a minor probability event. For uncontinuous or partly continuous problems, non-sampling methods are used. For example, perturbation methods [21] have been extensively used in various engineering fields [21,22,23]. In order to perform well, these methods should obey the inherent limitation that the uncertainty magnitude cannot be too large, both for the inputs and outputs (typically less than 10 percent). Another class of non-sampling methods includes the operator-based methods [24], which are actually manipulations of the stochastic operators in the governing equations. Like perturbation methods, they are also restricted to small uncertainties [25] and strongly dependent on the underlying operators. We previously developed adequate processing models to predict the device performance [26]. However, these approaches are not applicable to complex device models with different process deviation distributions.

A generalized polynomial chaos (GPC) method can be qualified, which is essentially a spectral representation in random space [27]. It exhibits fast convergence when the solution smoothly depends on the random inputs. This idea derives from polynomial chaos (PC), based on the theory of Wiener-Hermite polynomial chaos [28]. The Hermite polynomials aim to represent random processes as orthogonal basis and succeed in solving engineering problems [28]. The PC expansion is to construct a random variable with a desired distribution as a function of the given random variable. As a result, the GPC type of random variables depends on random inputs, following the Wiener-Hermite PC expansion. This approach, which provides not only high accuracy, but faster convergence rate, has also been successfully applied to many engineering problems, such as computational mechanics [29], diffusion [30], fluid flow [31] and heat conduction [32].

This work is devoted to the GPC-based stochastic modeling of MEMS beam structures. To quantify the effect of stochastic deviations in MEMS processing, the random inputs are finally turned into deterministic equations. Various process deviations are assumed to be mutually independent and discretized in the governing equations within stochastic domains. Sensitivity analysis is utilized to choose the initial critical factors. The doubly-clamped poly-beam has been testified to illustrate the accuracy of the GPC method. GaAs MMIC-based beams are modeled to predict the statistical features of the unknown parameters such as Young’s modulus and the residual stress. Verifications have been conducted by MC simulations as well as experimental tests by a Laser Doppler Vibrometer (LDV, MSV-400M2-20, Polytec Corp., Irvine, CA, USA).

## 2. Methodology and Algorithm

All process deviations are assumed to be represented by a vector x. Thus, the original device performance equations can be rewritten as in Equation (1) in a stochastic way rather than deterministically:

(1) 𝓛(x,t,θ;u)=f(x,t,θ) ,

where u=u(x,t,θ) is the performance solution, f(x,t,θ) is the source term. The differential operator 𝓛 generally involves differentiations in space/time and can be nonlinear. Appropriate boundary and initial conditions should be set before the simulation. The random parameter θ represents the introduction of uncertainties into process parameters via boundary conditions, initial conditions, material properties, etc. A simple approach is MC sampling, which involves generating independent but identically distributed random variables $\left\{\xi^{j}\right\}=\left[\xi_{1}\left(\theta_{j}\right),\ldots ,\xi_{n}\left(\theta_{j}\right)\right],\;for\;j=1,\ldots ,N,$ with the given number of realizations N. For each realization, the deterministic problem $𝓛\left(u^{j},\sigma^{j},P_{f}^{j};x,t,\xi^{j}\right)=0$ is solved to obtain the solution [33]. However, MC-based simulators are often complex and take appreciable computing time to be evaluated, so that the large number N of simulation runs required by MC is impractical. The performance solution u, which is regarded as a random process, can be expanded by the Wiener-Askey polynomial chaos as:

(2) $$u\left(x,t;\theta \right)=\;\overset{P}{\underset{i=0}{\sum }}u_{i}\left(x,t\right)\Psi_{i}\left(\xi \left(\theta \right)\right)\;.$$

Note that the infinite summation in Equation (2) has been truncated at the finite term P. The above representation can be considered as a spectral expansion in the random dimension θ, while the random trial basis $\left\{\Psi_{i}\right\}$ is the Askey scheme-based orthogonal polynomials. The total number of expansion terms is (P+1) which is determined by the dimension (n) of random variable ξ and the highest order (p) of the polynomials $\left\{\Psi_{i}\right\}$:

(3) $$\left(P+1\right)=\frac{\left(n+p\right)!}{n!p!}\;.$$

Upon substituting Equation (2) into the governing Equation (1):

(4) $$𝓛\left(x,t,\theta ;\overset{P}{\underset{i=0}{\sum }}u_{i}\Psi_{i}\right)=f\left(x,t,\theta \right)\;,$$

a Galerkin projection [28] of the above equation onto each polynomial basis $\left\{\Psi_{i}\right\}$ is conducted in order to ensure that the error is orthogonal to the functional space, spanned by the finite dimensional basis $\left\{\Psi_{i}\right\}$:

(5) $$‹𝓛\left(x,t,\theta ;\overset{P}{\underset{i=0}{\sum }}u_{i}\Psi_{i}\right),\Psi_{k}›\;=‹f,\Psi_{k}›,\;k=0,1,\ldots ,P.$$

By using the orthogonality of the polynomial basis, a set of (P+1) coupled equations can be obtained for each random mode $u_{i}\left(x,t\right)$, where i={0,1,…,P}. It should be noted that by utilizing the Wiener-Askey polynomial chaos expansion, the randomness in process inputs is effectively transferred into the basis polynomials. Thus, the governing device performance equations for the expansion coefficients $u_{i}$ resulting from above are deterministic. Discretizations in process inputs x and time t can be carried out by any conventional deterministic techniques, e.g., Runge-Kutta, to complex multi-physical MEMS problems for highly accurate solutions.

## 3. Problem Presentations and Analysis

MEMS process deviations mainly derive from the geometry, material properties and systematic errors. Although measures can be taken to reduce their influence, process deviations are totally inevitable. Advanced processing techniques and strictly environmental control aim at confining the geometric or material property deviations within desired ranges. Process deviations still occur randomly, giving rise to deviations from the original design. As the importance of input randomness has been highlighted above, the processing errors are treated herein as random events, along with process deviations as random variables. Illustrations on process deviations have been done with examples of MEMS beam structures in the following. Because of the uncertain nature of process deviations, the original deterministic systems are recast into stochastic systems which are handled by the GPC method and MC verification. When the corresponding polynomials for a given distribution can be built, it is best to employ these basis polynomials to produce the given distribution exactly [27]. Without loss of generality, the input processing parameters are normally distributed. As the analysis mainly focuses on in-plane movement and the resonant frequency, the influence of the air gap will be analyzed in our future work for performance like the capacitance.

### 3.1. The Doubly-Clamped Beam

As a basic element in MEMS, a doubly-clamped beam can be thought of to be an Euler-Bernoulli beam when the section is assumed to be a plane. The resonant frequency of the doubly-clamped beam underlies the majority of engineering designs. The differential equation for lateral oscillation can be expressed as Equation (6):

(6) $$\bar{EI}\frac{\partial^{4}z\left(x,t\right)}{\partial x^{4}}-\bar{\sigma A}\frac{\partial^{2}z\left(x,t\right)}{\partial x^{2}}=-\bar{\rho A}\frac{\partial^{2}z\left(x,t\right)}{\partial t^{2}}$$

where $\bar{EI}$ is the bending stiffness, $\bar{\rho A}$ is the linear density, $\bar{\sigma A}$ stands for the axial load, and z(x,t) is the displacement along the z-axis. Ignoring the residual stress, the resonant frequency of the doubly-clamped beam approximates as [34,35]:

(7) $$f_{i}=\frac{1}{2\pi }{\left(k_{i}l\right)}^{2}\sqrt{\frac{\bar{EI}}{\bar{\rho A}l^{4}}}$$

**Figure 1:**
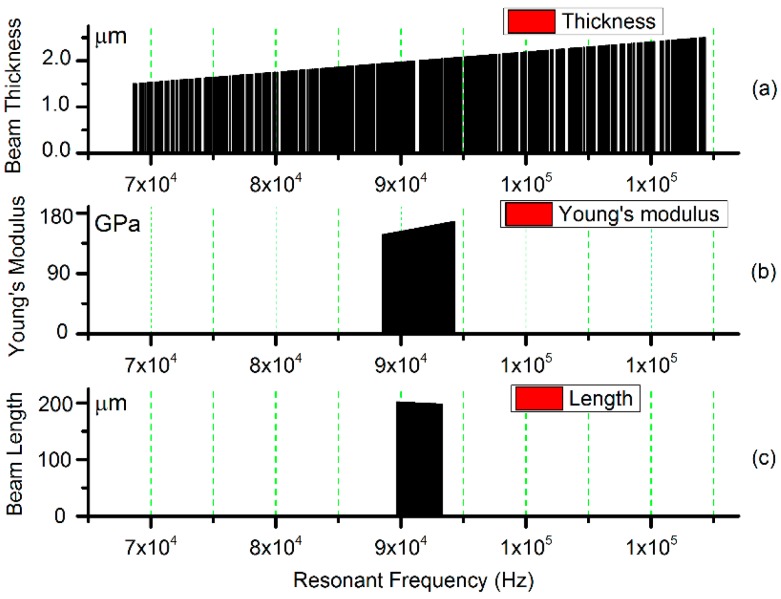
Sensitivity analysis of the resonant frequency for the doubly-clamped beam. (**a**) Beam thickness variable; (**b**) Young’s modulus variable; (**c**) Beam length variable.

**Figure 2:**
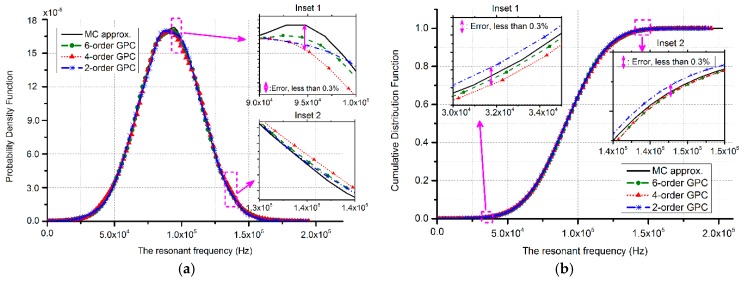
Statistical results of the resonant frequency for the doubly clamped beam approximated by three different orders of GPC methods and MC verifications. The marked insets demonstrate errors within 0.3%. (**a**) PDF; (**b**) CDF.

Here, $k_{i}l$ represents the coefficient of the *ith* mode of vibration. The first three values are $k_{1}l=4.730$, $k_{2}l=7.853$, $k_{3}l=10.996$, respectively. Process deviations are reflected in deviations of parameters E (Young’s modulus), h (beam thickness) and l (beam length). The sensitivity analysis can be conducted as Figure 1. The influence of beam thickness h in Figure 1a predominates over the other two factors, i.e., Young’s modulus and beam length in Figure 1b,c. Thus, it can be treated as one-dimensional stochastic problem. The width, length and Young’s modulus of the beam are fixed at 4 μm, 200 μm and 158 GPa, respectively while the thickness h changes randomly around initial value $h_{0}=2\;\mu m$. Assuming the distribution of h as $N\left(\mu_{h},\sigma_{h}\right)$, the resonant frequency can be approximated as:

(8) $$f_{i}=\sum_{i=0}^{P-1}\alpha_{i}\Psi_{i}\left(\xi \right)=\sum_{i=0}^{P-1}\alpha_{i}\Psi_{i}\left(h\left(\xi \right)\right)$$

where random variables ξ are the germs to construct random variable h, sharing similar distribution with h. The GPC method explained in Section 2 is applied to this case and verified with MC simulations (10,000 runs respectively). The results are plotted in a manner of probability density function (PDF) and cumulative distribution function (CDF), referring to Figure 2. As presented in Figure 2a, the results of 2-order GPC approximation can be a substitute for the MC method. The 4-order and 6-order PC approach MC simulations act with an error less than 0.3%, which can be obtained from the insets in Figure 2a,b. Therefore, for one-dimensional problems, 4-order PC approximations can satisfy the accuracy requirement.

### 3.2. GaAs MMIC-Based MEMS Beams

**Figure 3:**
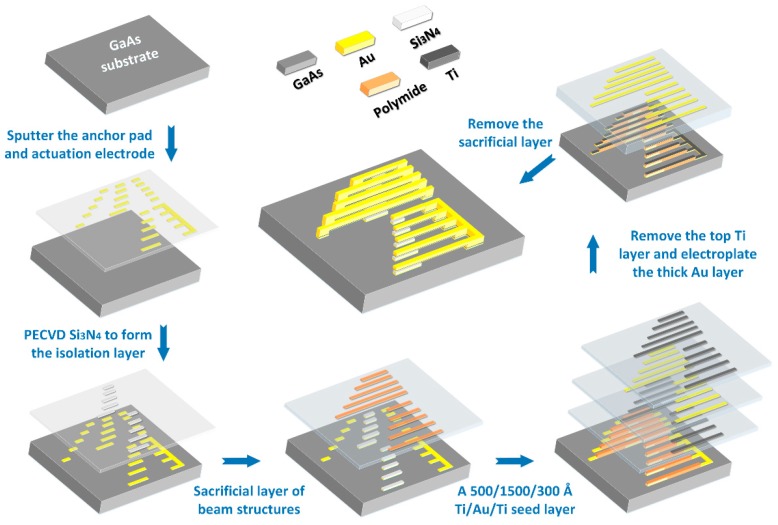
Processing steps of GaAs MMIC-based MEMS beams: the processing sequence is listed in the order of blue arrows.

**Figure 4:**
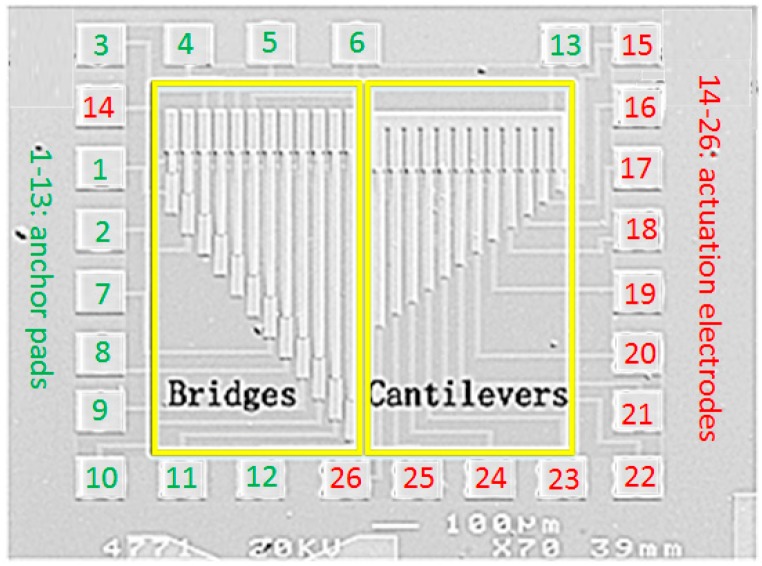
Topological SEM of GaAs MMIC-based MEMS beams: the left for bridge structures with the length ranging from 50 μm to 600 μm while the right for cantilevers with the length ranging from 50 μm to 380 μm. The spacing is 50 μm and 30 μm, respectively, for bridges and cantilevers. All the structures are 16 μm -width and 2 μm -thickness [36].

GaAs MMIC-based devices are significantly affected by Young’s modulus and the residual stress. These two properties of the GaAs MMIC-based MEMS films have been reported adequately in our group [30]. They may not only lead to failure of the micromachined devices by fatigue or environmental degradation, also alter expected devices performance by crazing or changing in shapes. Therefore, for reliability or yield improvements of these devices, it is necessary to predict and analyze the distributions of Young’s modulus and the residual stress in GaAs MMIC-based MEMS films. The resonant frequency of a cantilever beam, demonstrated in Equation (9), is related to the Young’s modulus, physical dimension, and material density. Parameters in Equation (9) are listed in Table 1. Experiments for the testing structures have been accomplished with processing procedures in Figure 3 and SEM structures in Figure 4 [36]:

(9) $$f_{cantilever}=0.16154\frac{h}{L^{2}}\sqrt{\frac{E_{e}}{\rho }}\;$$

**Figure 5:**
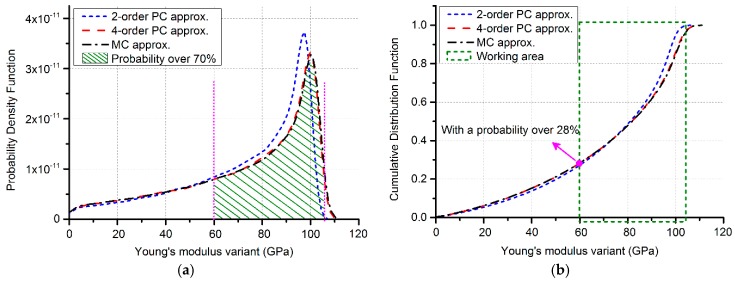
Statistical features of Young’s modulus: (**a**) PDF; the probability is over 70% while the horizontal axis ranges from 60 GPa to 105 GPa; (**b**) CDF; the intersection point takes a probability over 28% to evaluate Young’s modulus to 60 GPa.

The influence of beam width deviations can be ignored as it is less sensitive to process deviations compared with the thickness. The key element in Equation (9) turns out to be the beam thickness h by means of the sensitivity analysis. The value of beam frequency $f_{cantilever}$ is set to 25.2 kHz, according to its uniform distribution of testing results. The GPC method has been conducted on both the variables, beam thickness h and Young’s modulus $E_{e}$, where the parameter h is assumed to be normally distributed. Herein, the 2-order and 4-order GPC are chosen to compare with MC simulations (10,000 runs). Concentrated on the statistical features of Young’s modulus, plots of PDF and CDF are shown as Figure 5. For the illustrative purpose, beam thickness deviations are a bit exaggerated. Besides, there still exist deviations in beam frequency $f_{cantilever}$. As a result, the exceptional values should be excluded. The working area of Young’s modulus as the dashed area in Figure 5a and circled green box in Figure 5b, is assigned to be [60 GPa, 105 GPa].

Over 70% simulation data is included in Figure 5a, which means that the intersection in Figure 5b has a probability over 28% to evaluate Young’s modulus more than 60 GPa. The 4-order GPC approximation in Figure 5 has shown an appropriate match with MC verifications with the error less than 1% totally.

**Figure 6:**
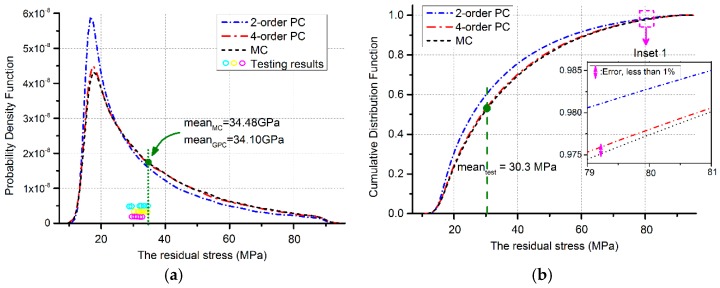
Statistical features of the residual stress: (**a**) PDF approximated by the GPC and MC method (100,000 runs), along with results calculated from testing of 6 samples at room temperature and atmospheric pressure, where the Cyan, magenta and yellow circles correspond to the beam structures with length of 250 μm, 200 μm and 150 μm, respectively; (**b**) CDF approximated by the GPC and MC method, with inset 1 for precision explanations and the marked mean value of testing.

The left part in Figure 4 is the bridge structures. Its resonant frequency is a function of both Young’s modulus and the residual stress, indicated as Equation (10). Here, $\sigma_{u}$ represents the effective residual stress while the rest parameters share the same meaning as Equation (9). Concluded from the statistical features of Young’s modulus, the mean value is deduced as 76.89 GPa. Thus, the residual stress $\sigma_{u}$ can be revised into a function of the parameter h, $\sigma_{u}=\sigma_{u}\left(h\right)$. Here the value of the resonant frequency $f_{bridge}$ equals 159.5 kHz, referring to its uniform distribution of testing results. Similarly, statistical properties of the residual stress can be obtained from its PDF and CDF plots in Figure 6a,b, with the help of the GPC and MC implementations. Several statistical features are listed in Table 2 to demonstrate the advantages of space / time-saving for the GPC methods compared with different MC simulation runs (Windows 7, Intel(R) Xeon(R) CPU E5-2630 v4 @ 2.20 GHz, 64.0 GB RAM). The mean value of the huge 100,000 MC samplings can be treated as a reference, to which the 4-order GPC shares an error about 1.10% but nearly 5 times faster. The testing results in Figure 6a locate in the range of $\left[\mu_{PC}-2\sigma_{PC},\mu_{PC}+2\sigma_{PC}\right]$, where $\mu_{PC}$ and $\sigma_{PC}$ are the mean and standard deviation of the residual stress for the 4-order GPC approximation. The errors in Figure 6 between 4-order GPC and MC method are less than 1% as the inset indicates in Figure 6b. Thus, a prediction can be made from Figure 6 that the probability of the residual stress to attain the mean testing value is about 54.3%:

(10) $$f_{bridge}=1.028\frac{h}{L^{2}}{\left(\frac{E_{e}}{\rho }\right)}^{1/2}{\left[1+0.295\frac{\sigma_{u}}{E_{e}}{\left(\frac{L}{h}\right)}^{2}\right]}^{1/2}$$

## 4. Discussion

The MEMS beam structures used in this study are simplified into one-dimensional stochastic systems, which make the high order GPC expansion unnecessary. Examples of the doubly-clamped poly-beams in Section 3.1 have demonstrated that the accuracy of 4- or 6-order between GPC and MC is over 99.7%. Section 3.2 has taken the advantage of the correlation between the cantilever and bridge structures to give the distributions of unknown parameters. Considering the exaggerated input deviations, Young’s modulus is tackled with a probability more than 28% while the error remains less than 1%. As a result of the sensitivity analysis, Young’s modulus and the resonant frequency are evaluated at determinate points in bridge structure analysis. The distribution of the residual stress is concluded as Figure 6. The mean value of the residual stress has undergone a left-drift because of the exaggerated random variable and the ignored parameter randomness in beam length, Young’s modulus, and the resonant frequency. Due to these factors, the simulation results experience an underestimated probability to attain the mean value of experimental tests. In this case the predicted probability should be higher than 54.3% to gain the experimental result. Nevertheless, the one-dimensional variable situation can act as an important index for MEMS device performance predictions, concerning to random process deviations.

## 5. Conclusions

This work has proposed a framework to model MEMS beam structures under process deviations. The modeling is accomplished by employing a GPC method to replace the role of brute-force MC simulations. Comparisons among the GPC, MC approximations, and experimental tests have been made to verify the accuracy of the GPC method with an acceptable error. It can be concluded that by proper selection of distribution terms and expansion orders, the GPC method can be utilized to replace the time/space-consuming role of MC simulations for more complex MEMS devices. Furthermore, suggestions can be obtained for compensation designs and yield improvement under processing uncertainties.

## Figures and Tables

**Table 1:** Relative parameters in Equation (9).

Parameters	Design Values ± Deviations
Beam length (L/μm)	170 ± 1
Beam thickness (h/μm)	2 ± 0.5
Material density ($\rho /g\cdot {\mathrm{cm}}^{-3}$)	19.2
Effective Young’s modulus ($E_{e}$)	$E/(1-\nu^{2})*$

* ν is the Possion’s ratio, and here it equals 0.42 for gold [12].

**Table 2:** Mean location of the residual stress and its standard deviation by GPC and MC.

	GPC		MC		
	2-Order	4-Order	100 Runs	1000 Runs	100,000 Runs
Mean value (GPa)	31.55	34.10	31.27	33.66	34.48
Error	8.50%	1.10%	9.31%	2.38%	--
